# Commercialization of the Xalkori Pediatric Multiparticulate Product Using Quality-by-Design Principles

**DOI:** 10.3390/pharmaceutics16081027

**Published:** 2024-08-01

**Authors:** Jeremy Bartlett, Natalie Culver, Xiang Zhang, Brett Waybrant, Hannah Sullivan, Logan Howell

**Affiliations:** 1 Pfizer, Eastern Point Road, Groton, CT 06340, USA; 2 Lonza, 63045 NE Corporate Place, Bend, OR 97701, USA

**Keywords:** pediatrics, multiparticulates, microspheres, dose flexibility, uniformity, commercialization, quality by design (QbD), design of experiments (DoE), process optimization, material-sparing approach, melt spray congeal (MSC), pellets, lipid multiparticulates, taste masking, volumetric dosator

## Abstract

A pediatric dosage form for crizotinib (Xalkori) was commercialized using quality-by-design principles in a material-sparing fashion. The dosage form consists of spherical multiparticulates (microspheres or pellets) that are coated and encapsulated in capsules for opening. The crizotinib (Xalkori)-coated pellet product is approved in the US for pediatric patients 1 year of age and older and young adults with relapsed or refractory, systemic anaplastic large cell lymphoma (ALCL) and unresectable, recurrent, or refractory inflammatory myofibroblastic tumor (IMT) that is ALK-positive. The product is also approved in the US for adult patients with non-small cell lung cancer (NSCLC) who are unable to swallow intact capsules. The lipid multiparticulate is composed of a lipid matrix, a dissolution enhancer, and an active pharmaceutical ingredient (API). The API, which remains crystalline, is embedded within the microsphere at a 60% drug loading in the uncoated lipid multiparticulate to enable dose flexibility. The melt spray congealing technique using a rotary atomizer is used to manufacture the lipid multiparticulate. Following melt spray congealing, a barrier coating is applied via fluid bed coating. Due to their particle size and content uniformity, this dosage form provides the dosing flexibility and swallowability needed for the pediatric population. The required pediatric dose is achieved by opening the capsules and combining doses of different encapsulated dose strengths, followed by administration of the multiparticulates directly to the mouth. The encapsulation process was optimized through equipment modifications and by using a design of experiments approach to understand the operating space. A limited number of development batches produced using commercial-scale equipment were leveraged to design, understand, and verify the manufacturing process space. The quality by design and material-sparing approach taken to design the melt spray congeal and encapsulation manufacturing processes resulted in a pediatric product with exceptional content uniformity (a 95% confidence and 99% probability of passing USP <905> content uniformity testing for future batches).

## 1. Introduction

### 1.1. Product Background

The crizotinib-coated pellet product is approved in the US for the treatment of relapsed or refractory, systemic anaplastic large cell lymphoma (ALCL) and unresectable, recurrent, or refractory inflammatory myofibroblastic tumor (IMT) that is ALK-positive. While the crizotinib-coated pellet product was developed for the pediatric patient population for patients 1 year or older, it is also approved in the US for adult patients with non-small cell lung cancer (NSCLC) who are unable to swallow the standard adult capsule formulation (crizotinib capsule, administered as intact capsules).

This pediatric formulation is a taste-masked, coated, spherical multiparticulate (microsphere, granule, or pellet). During the initial pediatric formulation development, the crizotinib API was evaluated as an oral solution and was found to have a very challenging palatability profile with both bitterness and burning/irritation. Formulation development of a clinical oral solution focused on excipients needed for taste masking, including extensive screening of sweeteners, flavors, and mouthfeel modifiers. Even with this focus on optimizing an oral solution formulation of crizotinib to mitigate the palatability challenges, it was not tolerated. This resulted in parts of a pediatric oncology study that used the oral solution being terminated due to the poor taste and tolerability of the oral solution [1,2]. The burning and irritation were linked to the drug being in solution and hitting the taste receptors in the mouth and throat.

A coated microsphere formulation, along with its dosing and administration options, was designed to minimize the amount of drug that would be in solution in the mouth to reduce the risk of poor taste and burning/irritation. The API was embedded within a wax-based multiparticulate to reduce the interaction of the API with the taste receptors within the mouth. A barrier coating was then applied to the multiparticulates as a means of further preventing the API from releasing in the mouth during dosing.

Dose flexibility was achieved by encapsulating three strengths, which can be combined to reach the target dose. The capsules (20 mg, 50 mg, and 150 mg) are not intended to be swallowed whole. Instead, the design for administration of the product was for capsule contents to be emptied directly into the patient’s mouth or emptied into a consumer-supplied oral dosing aid (e.g., spoon, medicine cup) and then to the patient’s mouth, followed by water. Dosing flexibility is achieved by combining the contents of capsules, as needed, to obtain the required target dose [3]. The product was not designed for sprinkling over food as this was seen as a potential risk to the taste-masking strategy (the API may dissolve out of the waxy multiparticulate over the time course of the food being consumed, thus defeating the taste-masking strategy of the dosage form). This coated multiparticulate formulation, designed to be dosed straight to the mouth, was accepted by the patient population, thereby overcoming the palatability challenges encountered with the oral solution. Multiparticulate technology has many advantages over more traditional formulation options and can be used to streamline product development for pediatric dosage forms.

### 1.2. Advantages of Multiparticulates

The pediatric patient population is underserved with respect to prescription medication, and the design of pediatric dosage forms is challenging due to children’s varied physiology and preferences as they mature [4,5]. Multiparticulate solid oral dosage forms have been identified as pediatric dosage forms that address many of the development and use challenges associated with pediatric oral liquids [4,5,6]. There are more chemical and physical stability challenges for oral liquids. Challenges in taste masking the active pharmaceutical ingredient often require the use of a diverse range of excipients, preservatives, and flavors (and then regional flavor preferences) [4]. There is a strong encouragement from regulatory bodies for preservative-free pediatric formulations [6]. Oral solids are often preferred over oral liquids for pediatric dosage forms because they do not contain preservatives [7]. Inaccurate dosing is more frequent with liquid formulations than solid dosage forms [5]. Oral liquids have higher costs to transport and store, especially if the liquid requires refrigeration [8]. The future of pediatric oral formulations is with single-use, preservative-free, taste-masked dosage forms, including multiparticulates [6]. While the literature is clear that solid oral dosage forms designed with pediatrics in mind are a preferred route, their wide acceptance by healthcare practitioners has yet to be seen [9].

Multiparticulates can be formulated into various final drug product presentations, from the more straightforward sprinkle capsules, stick-packs, or sachets to being incorporated into orally disintegrating tablets (ODTs) or compressed tablets. They can also be combined with a liquid and dosed as an oral suspension and, if designed appropriately, are often suitable for enteral feeding tube administration when combined with a liquid. This versatility of dosing and administration options is another advantage of flexible oral solids compared to traditional liquid formats, which are primarily dosed via an oral syringe to the mouth.

Multiparticulates consist of multiple small units on the order of 100 µm to around 3 mm that are easy to swallow. Sprinkled (multiparticulates) were generally more acceptable over oral liquids in children ranging from 5 months to 16 years of age, although texture and viscosity of the vehicle, if used, were found to have an impact on acceptability [4]. Each small unit contains the same functionality, such as taste masking, as the overall dosage form. Due to their particle size and content uniformity, multiparticulate dosage forms provide the swallowability and the dosing flexibility needed for the pediatric population. Dose flexibility, needed for clinical trials and for pediatric populations that require multiple strengths, is obtained by varying the quantity of microspheres delivered to the patient.

When taken orally, multiparticulates generally disperse freely in the gastrointestinal tract (GI), maximize absorption, and minimize side effects [10,11]. Multiparticulate systems give a more reproducible drug release, especially for targeted delivery in the small intestine or colon, due to their transit pattern through the GI tract. For example, large monoliths such as tablets have variable transit times and can skip over the haustral ridges of the colon without contacting the entire mucosal wall, while multiparticulates spread out through the length of the GI tract and progress with slower transit time through the colon [12]. Multiparticulates can provide a variety of different release profiles through different formulation approaches, such as immediate release, taste masking, modified release, and enteric protection.

Age-appropriate pediatric formulations are needed for patient compliance in children. Pediatric compliance has been reported to have an average of 58% adherence, with one of the key contributing factors being the pediatric dosage form and palatability [13]. The acceptability of pediatric multiparticulate dosage forms is well-known in the literature [6,14,15,16,17]. In one study, multiparticulates with a median diameter D50 of 263 µm were found to be more swallowable by children ages 2–8 years than a minitablet of 2 mm in diameter, especially among the younger age brackets [14]. There has been a shift to leveraging flexible oral solids (multiparticulates) for pediatric medications versus traditional oral liquids [6]. With these advantages in mind, a multiparticulate approach was selected to enable the taste-masking and dose flexibility requirements for the crizotinib pediatric product.

### 1.3. Problem Statement

The pediatric patient population for systemic ALCL and unresectable, recurrent, or refractory IMT that is ALK-positive is small, and a limited number of clinical studies were required for this product. Therefore, the manufacturing process needed to be optimized using a small number of development and clinical batches. To minimize material usage, the design of experiments (DoE) following a QbD approach [18] was used to reduce the number of batches needed to define and verify the manufacturing process space. For example, instead of running a full batch quantity at each DoE condition, multiple process conditions were explored with a single batch size quantity on full-scale equipment. This significantly reduced the material required; for example, one encapsulation DoE to define the process space was completed with around 2 kg of coated microspheres.

## 2. Materials and Methods

### 2.1. Equipment

The equipment used in this manufacturing process includes traditional bin blending equipment (Bin Blender, Meto, Franklin Lakes, NJ, USA), a gravimetric feeder (K-tron, Coperion, Sewell, NJ, USA), extrusion (ZSE-27, Leistritz, Allendale, NJ, USA) and melt spray congeal with a custom rotary atomizer (Lonza [19], Bend, OR, USA), screening (Turbo Screener, Freund Turbo Corporation, Marion, IA, USA), fluid bed coating with bottom spray Wurster column (Lonza, Bend, OR, USA), encapsulation with custom vacuum dosator (Modified IMA Zanasi Plus 70E with custom volumetric dosator [20], Bologna, Italy), and capsule weight sorting (KKE 1700, Bosch, Waiblingen, Germany). Lonza designed and built the manufacturing equipment required to enable the GMP manufacture of this product. All equipment is installed at the Lonza Bend GMP manufacturing facility in Bend, OR, USA. Pfizer and Lonza collaborated on the design of the manufacturing process.

### 2.2. Materials

The active pharmaceutical ingredient (API) crizotinib was sourced from Pfizer. The lipid multiparticulate core comprises the API embedded in a matrix of stearyl alcohol (Kolliwax^®^ SA, BASF Pharma, Florham Park, NJ, USA) and poloxamer P407 (Kolliphor^®^ P 407, BASF Pharma, Florham Park, NJ, USA). The coating consists of a high gloss aqueous sugar film system (Opadry^®^ SGR, Colorcon, Harleysville, PA, USA). The coated microspheres are encapsulated in hard gelatin capsules (Coni-snap^®^, Lonza, Basel, Switzerland).

### 2.3. Design of Experiments

Experimental design and data analysis were performed in Design Expert (Stat-Ease^®^, Minneapolis, MN, USA) version 12.0.9.0 and JMP^®^ (JMP^®^ Statistical Discovery, Cary, NC, USA) version 17.2.0.

## 3. Multiparticulates Manufacturing

### 3.1. Manufacturing Process

A manufacturing process to produce crizotinib-coated microspheres was commercialized using quality-by-design principles in a material-sparing fashion. Crizotinib-coated microspheres are manufactured in a multi-step process: (1) blending of the raw materials; (2) melt extrusion, forming a liquid suspension (melt) with the API; (3) spray congealing of the melt to form uncoated microspheres; (4) screening to select the desired particle size; (5) film coating of the microspheres. The coated microspheres are then encapsulated in capsules for opening.

### 3.2. Melt Spray Congeal

**Figure 1:**
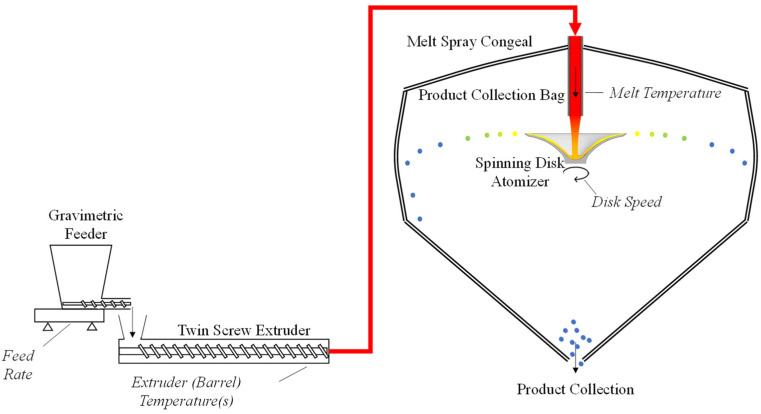
Process flow diagram of the melt spray congeal process. The red designates the heated path the melt follows from the extruder to the atomization disk. The cartoon depicts the droplets atomizing from the disk and cooling to the point of congealing, indicated by the color changing from yellow to blue, prior to impacting the collection bag.

The uncoated lipid multiparticulates, consisting of the active pharmaceutical ingredient embedded in a lipid matrix with a dissolution enhancer, are manufactured by melt spray congealing (MSC). Melt spray congealing refers to the gravimetric feeding, melt extrusion, and spray congealing unit operations, as shown in Figure 1. Prior to introduction to the gravimetric feeder, the API is blended with excipients using a bin blending process to pre-mix the materials prior to introduction to the extruder. The lipid excipients are melted in the extruder, and the crystalline API is suspended in the melt. This suspension is conveyed to a rotary atomizer, which produces uniform droplets. The droplets congeal in flight prior to collection in the product collection bag. The resulting lipid multiparticulates are spherical particles with crystalline API embedded in the lipid matrix.

**Figure 2:**
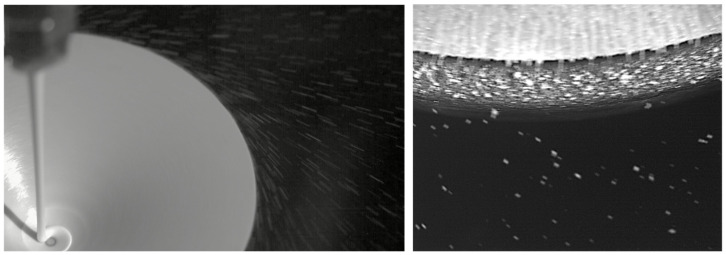
High-speed video footage of the crizotinib melt being dispensed onto the spinning disk and atomized on the rotary atomizer.

**Figure 3:**
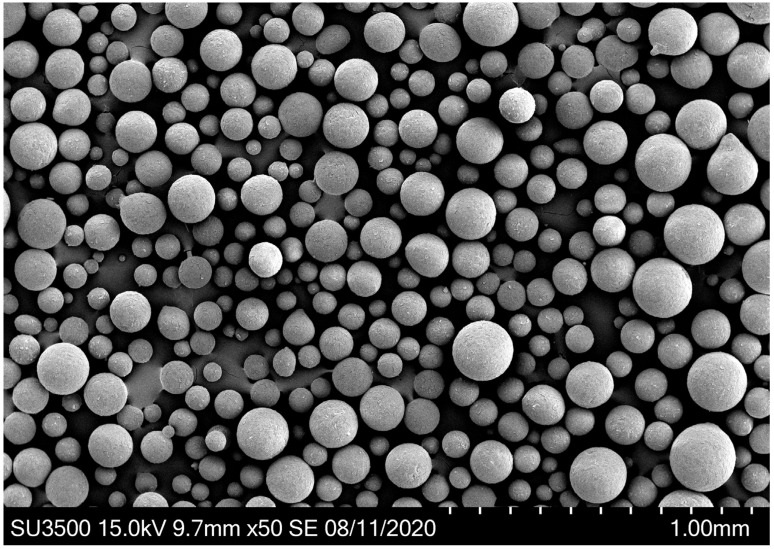
Representative particle morphology for crizotinib lipid multiparticulates produced via MSC.

Rotary atomization is utilized in the MSC process as it has the ability to process viscous, highly loaded suspensions and produce droplets with a tight size distribution. Atomization dynamics for suspensions have been studied, and particle size models have been proposed in the literature [21]. These models were leveraged during process development to design a robust atomization operating space. An example image of the rotary atomization is provided in Figure 2 and Supplemental Video S1. Particles produced as a result of the MSC process are highly spherical, as illustrated in Figure 3.

**Figure 4:**
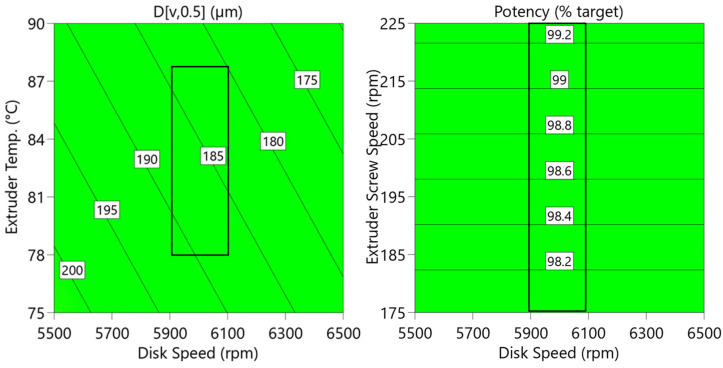
Particle size (D[v,0.5] (µm)) (left) as a function of the two statistically significant process parameters. Potency (right) as a function of the one statistically significant process parameter (extruder screw speed). The black boxes in both figures denote the narrowed operating space to ensure consistent microsphere particle size. Note: Supplement Table S1 reports the study design.

The MSC process was optimized to yield highly uniform microspheres with a high loading of API. The API loading in the lipid multiparticulate was maximized at 60% for this formulation to enable the encapsulation of a wide range of dose strengths using a common intermediate microsphere. Process optimization studies utilized material-sparing DoE to understand the impact of four MSC process parameters on microsphere particle size and potency uniformity (powder reed rate into the extruder, extruder screw speed, extruder temperature, and spinning disk speed); see Supplement Table S1. An initial fractional factorial DoE indicated that extruder temperature and disk speed had a statistically significant impact on particle size, while only extruder screw speed had a statistically significant impact on potency. Further optimization studies focused on these process parameters, and a robust operating space was identified that resulted in consistent microsphere particle size and potency, as illustrated in Figure 4. Potency values showed little variation across the explored design space. While the observed particle size variance across the explored range was deemed acceptable from a product quality standpoint, the intended operational space was narrowed to ensure microspheres of a consistent and uniform size were produced. The intended operational space is denoted by a black box on both plots in Figure 4.

**Figure 5:**
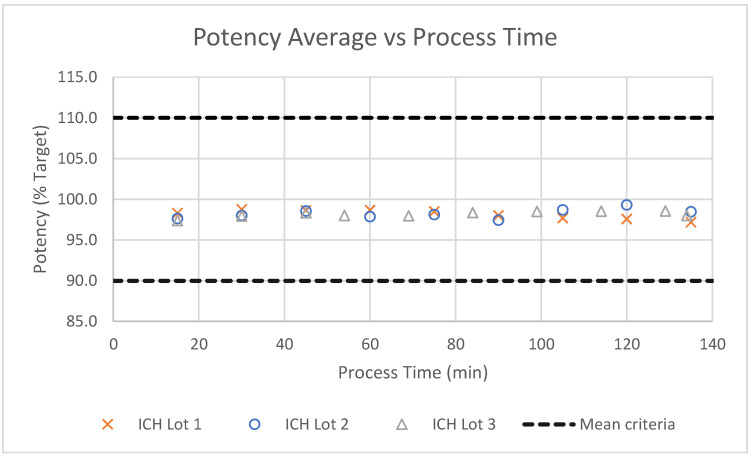
Potency across three registrational stability batches of uncoated microspheres as a function of process time.

**Figure 6:**
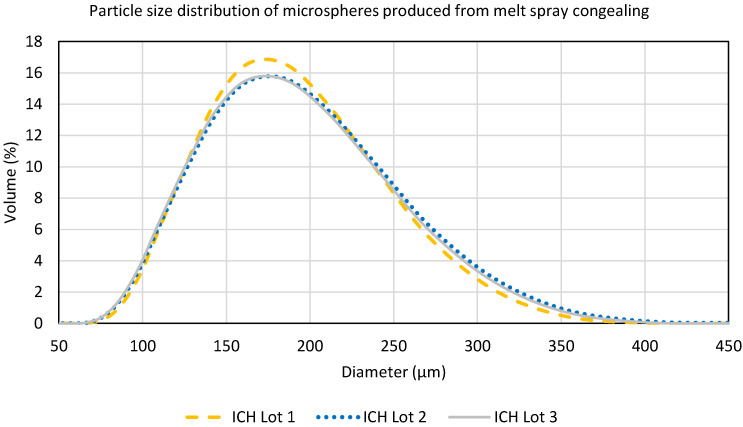
Particle size distribution by laser diffraction for registrational stability lots of uncoated microspheres produced via melt spray congealing prior to screening.

The uniformity of the MSC process was tracked as a function of process time for registrational stability batches (denoted ICH), as shown in Figure 5. The potency of the uncoated microspheres was consistent for the duration of the batch, illustrating the high degree of uniformity throughout the MSC process. Additionally, particle size statistics measured for each batch prior to screening indicate that the atomization process was also very consistent, as shown in Figure 6 and Table 1.

### 3.3. Fluid Bed Coating

Following the MSC process and screening, a barrier coating to enhance taste masking is applied to the lipid microsphere via fluid bed coating. The coated microspheres represent the final blend prior to encapsulation. Based on the uniformity data generated during process development, the process validation strategy (PPQ) focused on the final blend uniformity of the coated microspheres prior to encapsulation. The tabulated data are presented in Table 2, demonstrating that the coated microspheres are a highly uniform intermediate that enables the downstream encapsulation strategy.

## 4. Encapsulation

Encapsulation utilized a custom [20] volumetric dosing encapsulator to provide gentle handling of the coated microspheres and to achieve excellent capsule uniformity of dosage units (UDU). Three different strengths were produced to achieve the required dose flexibility for the pediatric population. During product development, a combination of equipment upgrades and statistical design of experiment batches was used to optimize the encapsulation process.

### 4.1. Equipment Upgrades

In a vacuum dosator system, a dosing tube of a consistent diameter is utilized to set a fixed product volume. Accurate volumetric dosing with a vacuum dosator requires consistent removal of excess product from the tip of the dosator. Vacuum pulls material into the dosing tube and holds it there until the excess material can be removed from the tip of the dosing tube.

**Figure 7:**
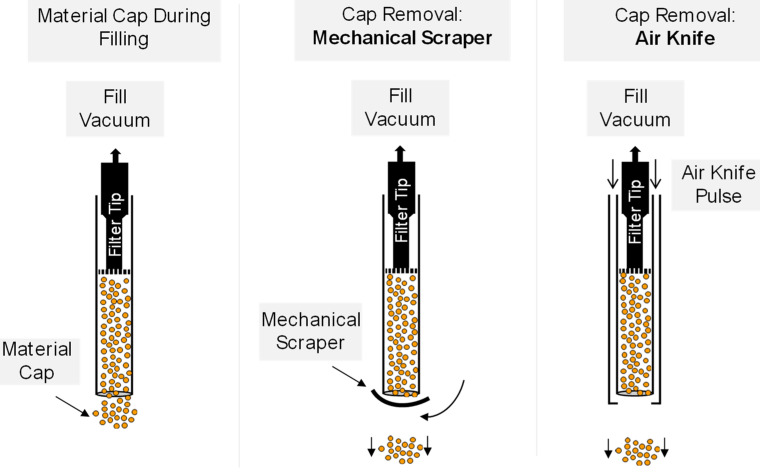
Mechanical scraper compared to air knife methods of removing the material cap.

Early in the product development process, the mechanism used to remove the excess material was a mechanical scraper. The mechanical scraper provided adequate fill weight uniformity, but there was room for improvement as the equipment set-up was difficult and significantly affected the filling uniformity. The equipment was modified to utilize a jet of air (air knife) to remove the excess material instead of the mechanical scraper. The differences between the two configurations are shown in Figure 7. This equipment upgrade reduced the fill weight variability and avoided many issues with inconsistent assembly of the mechanical scraper.

**Figure 8:**
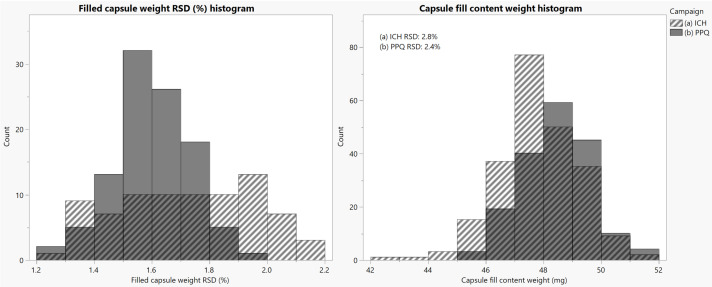
Left: Filled capsule weight RSD from the in-process control sampling for the (a) ICH campaign (mechanical scraper used) compared to (b) PPQ campaign (air knife used). Data are summarized for three batches of each campaign. Right: Capsule fill contents from extended uniformity of dosage units for the (a) ICH campaign (mechanical scraper used) compared to (b) PPQ campaign (air knife used).

A comparison between the Registration Stability (referred to as ICH batches in the following figures) campaign manufactured with the mechanical scraper and the Process Performance Qualification (PPQ) campaign manufactured with the air knife set-up is shown in Figure 8. Filled capsule weight RSD (Figure 8, left) summarizes in-process samples taken during the encapsulation batch every 15 min. In total, 72 capsules are weighed, and the RSD is reported to compare against a process limit. Capsule fill content weight (Figure 8, right) summarizes the fill mass of coated microspheres from extended uniformity of dosage units sampling. The authors use the term extended uniformity to describe the statistical sampling plan for demonstrating the uniformity of dosage units [22]. This technique consists of taking stratified samples of three capsules from pre-determined locations across a batch. The data in Figure 8 are a summary of the three ICH batches overlaid with the three PPQ batches. For both the filled capsule weight RSD and the capsule fill content, the air knife upgrades on the equipment significantly reduced the variability within the capsule population and improved the filling process.

**Figure 9:**
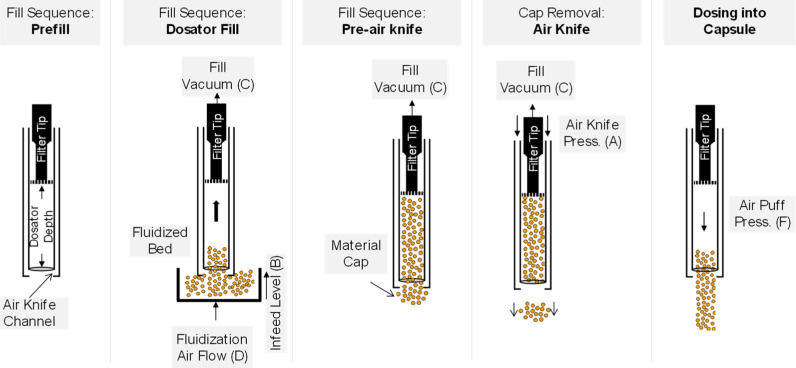
Volumetric dosing with a vacuum dosator. Process parameters are labeled and described in the text.

The encapsulator filling mechanism with the air knife upgrades is shown in Figure 9. The process is controlled through six process parameters: (A) air knife pressure, (B) infeed level, (C) fill vacuum, (D) fluidizing air flow rate, (E) fill speed, and (F) air puff pressure. In this process, coated microspheres are fed into a fluidized bed. The microspheres are maintained at a certain height, the infeed level (B). The level of fluidization is maintained through the fluidizing air flow rate (D). To start the encapsulation cycle, the dosator is filled by the fill vacuum parameter (C). The vacuum pulls material from the fluidized bed into the dosing tube. An air knife (A) utilizes compressed air to remove the excess material from the tip of the dosator. The coated microspheres are expelled from the dosator and into the capsule by mechanical actuation of the filter tip followed by a small burst of air called the air puff pressure (F). The encapsulation machine runs at a specified rate, fill speed (E), in capsules per hour.

Accurate volumetric dosing with a vacuum dosator requires consistent removal of excess product from the tip of the dosator. The encapsulation process development centered around understanding how the process parameters affected the removal of the excess material cap.

### 4.2. Process Space Determination through Statistical Design of Experiments

A limited number of development batches were used to identify the process space for encapsulation. Design of experiments (DoE) was leveraged to efficiently explore the effect of multiple process parameters on the resulting filled capsule weight variability for all three strengths. The 20 mg strength is the most challenging to encapsulate due to the low target fill weight of 47 mg. Reducing fill content weight variability for this low fill weight is difficult because the ratio of the dosator tip surface area to the dosator volume is the largest of the three strengths. Therefore, the 20 mg strength is the focus of this discussion.

In these studies, the filled capsule weight relative standard deviation (RSD) was used as the main response variable. While filled capsule weight RSD is not a critical quality attribute (CQA) for this product, the uniformity of dosage units (content uniformity by HPLC for the 20 mg strength) is a CQA. Filled capsule weight RSD was used as a surrogate response variable in the DoEs instead of content uniformity by HPLC to reduce the testing burden.

A 19-run I-optimal design (DoE Study 1) was conducted to evaluate all of the main effects and some of the two-way interaction effects for the following six factors: (A) air knife pressure, (B) infeed level, (C) fill vacuum, (D) fluidizing air flow rate, (E) fill speed, and (F) air puff pressure; see Supplement Table S2. The process parameters are shown on the encapsulation diagram in Figure 9 and explained in the text above. Material usage was minimized by running the encapsulator for only enough time to reach a steady state and to take a sample of 300 capsules for analysis. In this way, 19 run conditions were explored with approximately 2 kg of coated microspheres.

The filled capsule weight RSD for all of the experimental conditions explored in DoE Study 1 ranged from 1.73% to 2.83%. It was preferred to achieve an RSD of not greater than 2.50% to ensure desirable content uniformity of future batches (development RSD target). Since some of the conditions from DoE Study 1 exceeded this development RSD target, and DoE Study 1 pointed towards a more optimal processing space, additional development work was warranted. For further optimization, a 16-run response surface design (DoE Study 2) was performed to evaluate the three statistically significant process parameters from DoE Study 1: (A) air knife pressure, (B) infeed level, and (C) fill vacuum. The factors for this DoE are reported in Supplement Table S3. Additionally, the target settings and ranges for the parameters were shifted from the initial study to achieve a more optimal design space.

**Figure 10:**
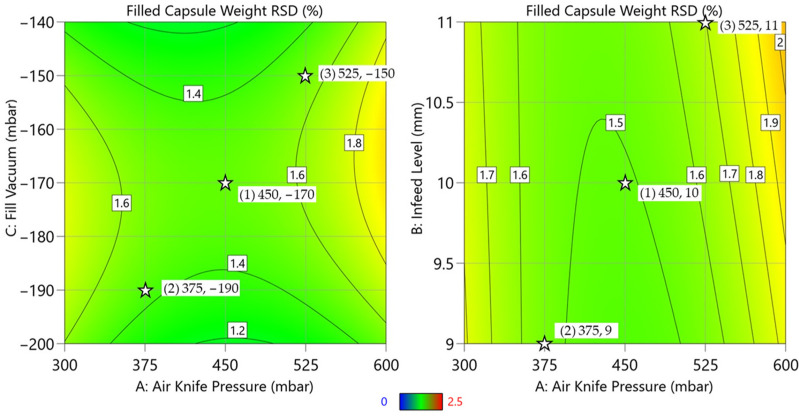
Contour plots for filled capsule weight RSD (%) (shown as values on the contour lines; color spectrum from blue (0%) to red (2.5%) for the RSD values is shown at the bottom of the figure) for the statistically significant two-way interaction effects of (1) AC: air knife pressure and fill vacuum (left) and (2) AB: air knife pressure and infeed level (right) based on DoE Study 2. The stars denote additional process exploration, as explained below in Section 4.3. Note: DoE Study 2 design and the results fitting and analysis are reported in Supplement Tables S3 and S4.

For DoE Study 2 with the optimized design space, the filled capsule weight RSD results ranged from 1.26% to 1.86%, with all conditions meeting the development RSD target of not greater than 2.50%. Additionally, the maximum predicted filled capsule weight RSD from the contour plot (Figure 10) is 2.10%, which is well within the limit of not greater than 2.50%. All three parameters studied in DoE Study 2 affected filled capsule weight RSD. Both (A) air knife pressure and (C) fill vacuum showed statistically significant quadratic effects (A^2^: *p*-value = 0.005; C^2^: *p*-value = 0.02), respectively. Also, there were two statistically significant two-way interaction effects: (1) AC: air knife pressure and fill vacuum (*p*-value < 0.001) and (2) AB: air knife pressure and infeed level (*p*-value = 0.014), as illustrated in Figure 10 and Supplement Table S4.

The interaction effect identified in the statistical model between (A) air knife pressure and (C) fill vacuum can be tied back to the physical situation occurring at the tip of the volumetric dosator. During cap removal (Figure 9), the fill vacuum is continuously pulling the microspheres into the dosator to keep them from dropping out. The air knife pulse must overcome the fill vacuum in order to remove the excess material (cap) from the tip of the dosator. There are scenarios where either not enough or too much material will be removed from the tip of the dosator with certain combinations of fill vacuum and air knife pressure set-points, increasing the variability in the fill contents (RSD). For example, a more negative fill vacuum setting and low air knife pressure may leave excess material on the tip of the dosator because the air knife pressure cannot overcome the fill vacuum.

In order to confirm the validity of using filled capsule weight RSD as a surrogate for content uniformity by HPLC, USP <905> methodology [23] was used to evaluate the content uniformity of the centerline and highest RSD condition from DoE Study 2. Filled capsule weight RSD correlates well with the final content uniformity results, as shown in Table 3. Both the centerline and highest RSD conditions passed the USP <905> content uniformity acceptance value upper limit of 15.

The DoE results were used to designate the criticality of the process parameters (either critical or non-critical) for the encapsulation process to define the control strategy. The development of a drug product manufacturing process requires identifying and assessing the impact of process parameters on critical quality attributes (CQAs) of the drug product, and a control strategy must be designed to control, at minimum, those process parameters that impact a critical quality attribute [18]. In practice, it is necessary to evaluate the criticality of process parameters in order to designate critical process parameters. A criticality designation assessment method was used to evaluate both the statistical and practical significance of encapsulation process parameters to determine critical process parameters [24]. For the encapsulation process, the focus of the DoE looked at the impact of the process parameters on the CQA of content uniformity.

DoE study 2 found that air knife pressure, infeed level, and fill vacuum had a statistically significant impact on filled capsule weight RSD (the surrogate for the CQA content uniformity). However, statistical significance alone does not indicate practical significance and, therefore, cannot be used solely to determine the criticality of the process parameter. A criticality assessment approach [24] was applied to determine the practical significance of the three statistically significant factors. The Z-score analysis of the DoE data was calculated to be 10.2, which is greater than the lower critical value of 6. Therefore, none of the three statistically significant parameters (air knife pressure, infeed level, and fill vacuum) were practically significant for content uniformity of 20 mg strength within the design space. As this work showed that these encapsulation process parameters do not practically impact the CQA of uniformity, the non-critical designation of these parameters was appropriate.

To confirm the results of the DoE, another batch, the process demonstration batch described below, with more material allocated to each run condition, was manufactured to demonstrate excellent content uniformity when the process was operated at the edges of the operating space.

### 4.3. Process Demonstration

**Figure 11:**
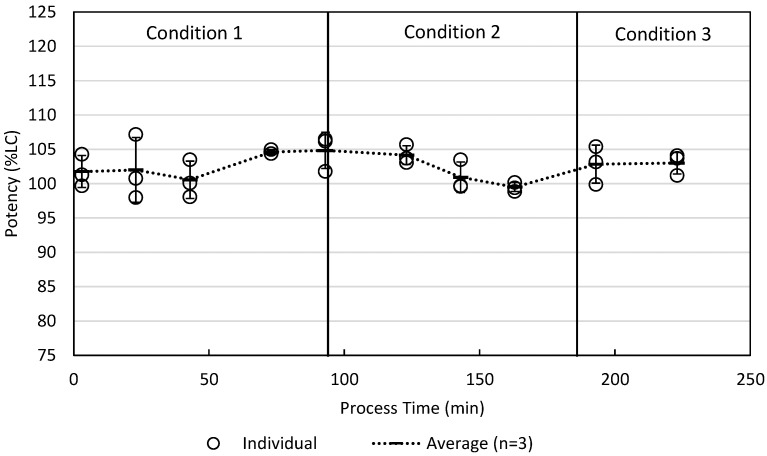
Extended uniformity of dosage units across the demonstration batch among three different processing conditions.

A process demonstration batch was manufactured, operating each third of the batch at different processing conditions, as outlined in Table 4. The first third of the batch was operated at target conditions (condition 1). The middle third of the batch was operated at a process space edge (condition 2), where infeed level, fill vacuum, and air knife pressure were set at the process control limits on one side of the process space. The last third of the batch was operated at a process space edge (condition 3), where infeed level, fill vacuum, and air knife pressure were set at the process control limits on the opposite side from condition 2 (reference Figure 10 for a comparison against the DoE process model). A summary of the in-process control test filled capsule weight RSD is shown in Table 4. This batch demonstrated that the operating space edges produced an acceptable product with good content uniformity (see Table 4 and Table 5 and Figure 11).

### 4.4. Process Validation

The processing space identified with the design of experiments development batch and confirmed with the process demonstration batch was used to manufacture three PPQ batches. The batches demonstrated excellent uniformity of dosage units, as tested by ASTM E2810 standard [25], with a 95% confidence and 99% probability of passing USP <905> content uniformity testing for future batches (Table 6).

## 5. Uniformity Discussion

**Figure 12:**
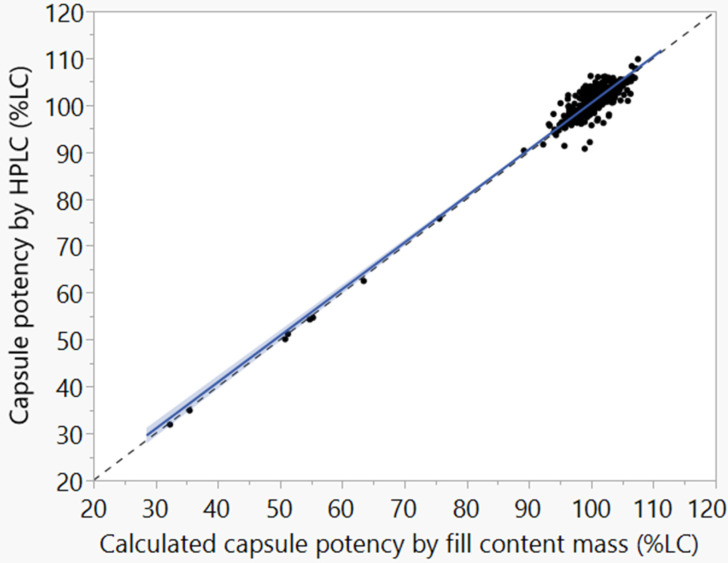
Capsule fill content uniformity for the ICH and PPQ campaigns, demonstrating the tight correlation between the fill content mass (X) and capsule assay (Y). Note that less than 2% of the capsules were less than 85 %LC and would be removed from the batch in the weight sorting process. Blue solid line: Y = 1.234 + 0.9921 × X (R^2^ = 0.943); Black dashed line: Y = X for reference.

The uniformity of the microspheres produced in this process is best demonstrated by Figure 12, which demonstrates that the relationship between the capsule fill mass (X-axis) and measured potency (Y-axis) is strongly correlated across a wide range of capsule fill masses. During capsule testing, the fill content mass for each capsule was weighed prior to assay. By calculating the expected potency based on the fill content mass and comparing it to the measured potency by HPLC, the two measurements were found to be equivalent, with a difference of less than 0.5% (90% confidence interval of the difference is [−0.59%, −0.31%]) which is well within the HPLC measurement uncertainty.

Another way to assess the microsphere uniformity is to calculate the weight-corrected potency for each capsule. This was performed by dividing the capsule HPLC assay value by the fill content mass to calculate the potency of the microspheres within each capsule. The weight-corrected assay standard deviation was 1.74 %LC across the entire data, which is indicative of highly uniform microspheres. Industry best practices target a standard deviation of less than 3.0% LC for blend uniformity, which would be sampled prior to standard tableting activities [22].

Even with a challenging low fill content mass of 47 mg, excellent product quality was obtained throughout the manufacturing process. This highlights the successful process design strategies utilized to evaluate the manufacturing process. A uniform product was manufactured at each step and was maintained through to the final product.

## 6. Conclusions

To summarize, a robust commercial control strategy was developed to enable a flexible dosing regime for the crizotinib-coated pellet product. This control strategy was developed in a material-sparing fashion using QbD principles. Heavy utilization of statistical designs of experiments (DoEs) allowed for rapid and efficient optimization of the melt spray congeal and encapsulation unit operations.

The flexible-dose presentation to the patient in multiple strength capsules for the opening was enabled by utilizing a common high-drug-load drug product intermediate. The drug product intermediate was manufactured using melt spray congeal followed by fluid bed coating. These unit operations were optimized to ensure particle size and potency uniformity were achieved.

The encapsulation process for the coated granules utilized a custom volumetric dosator to ensure a consistent fill mass with minimized variability was achieved across a range of dose strengths. The process parameters utilized for encapsulation were optimized through multiple rounds of material-sparing DoEs.

Following the optimization of the unit operations, excellent content uniformity was achieved for the crizotinib-coated pellet product. The process validation demonstrated a high degree of statistical confidence that future batches of all three capsule strengths would be capable of passing USP < 905> uniformity of dosage unit criteria.

The discussed coated multiparticulate technology represents an ideal platform to streamline pediatric drug product design, development, and commercial supply. Pediatric-specific formulations are often more challenging to develop, representing a higher technical barrier to achieving the target product profile. This higher technical barrier can add to the challenges of ensuring pediatric-specific formulations are developed in a timely manner. By focusing on streamlining and optimizing the process using multiparticulates, development cycle times can be reduced, which ultimately reduces the time required to get pediatric medicines to the patients who need them most.

## Figures and Tables

**Table 1:** Particle size summary statistics by laser diffraction for registrational stability lots of uncoated microspheres produced via melt spray congealing prior to screening.

Sample	D(v 0.1) µm	D(v 0.5) µm	D(v 0.9) µm	Span
ICH Lot 1	121.6	182.7	270.5	0.8
ICH Lot 2	122.7	184.8	273.5	0.8
ICH Lot 3	125.1	183.2	265.2	0.8

**Table 2:** Blend uniformity (10 locations, n = 3 from each location) of the coated microspheres for each process validation lot.

Sample	Average, %LC	Standard Deviation, %LC
PPQ Lot 1	100.3	0.5
PPQ Lot 2	99.6	0.3
PPQ Lot 3	99.6	0.5

**Table 3:** Content uniformity results by USP <905> compared to filled capsule RSD for encapsulation DoE Study 2.

Sample/Run Condition	Filled Capsule Weight RSD (n = 300, %)	Capsule Label Claim RSD (n = 20, %LC) by HPLC	Content Uniformity Acceptance Value by USP <905>
Centerline conditions	1.42	1.9	4.4
Highest RSD condition	1.86	2.6	7.7

**Table 4:** Process demonstration batch processing parameters and resulting filled capsule weight RSD (average from in-process control test of 72 capsules sampled every 15 min).

Run Condition (Numbered in Figure 10)	Infeed Level (mm)	Fill Vacuum (mbar)	Air Knife Pressure (mbar)	Filled Capsule Weight RSD (%)
Condition 1 (target)	10.0	−170	450	1.9
Condition 2 (operating edge)	9.0	−190	375	1.9
Condition 3 (operating edge)	11.0	−150	525	2.0

**Table 5:** Process demonstration batch extended uniformity of dosage units by ASTM E2810 [25], with a 95% confidence and 99% probability of passing USP <905> for future batches.

Lot	Overall Mean (%LC)	Individual Potency Range (%LC)	Between Location Std. Dev (%LC)	Within Location Std. Dev (%LC)	Pass/Fail ASTM E2810 (95%/99%)
Process Demonstration	101.2	95.8–107.2	2.0	2.9	Pass

**Table 6:** PPQ batches extended uniformity of dosage units by ASTM E2810 [25], with a 95% confidence and 99% probability of passing USP <905> for future batches.

Lot	Overall Mean (%LC)	Individual Potency Range (%LC)	Between Location Std. Dev (%LC)	Within Location Std. Dev (%LC)	Pass/Fail ASTM E2810 (95%/99%)
PPQ Lot 1	101.6	95.9–106.0	1.4	2.2	Pass
PPQ Lot 2	102.5	94.2–109.8	1.5	2.7	Pass
PPQ Lot 3	101.3	95.7–106.3	1.3	2.7	Pass

## Data Availability

The datasets presented in this article are not readily available due to the confidential nature of pharmaceutical product development.

## Supplementary Materials

The following supporting information can be downloaded at: https://www.mdpi.com/article/10.3390/pharmaceutics16081027/s1, Video S1: rotary atomization_crizotinib development MSC batch.avi. Supplement Table S1—Run conditions, MSC DoE (10 conditions/runs). The factors studied and description of study runs in Figure 4; Supplement Table S2—Run conditions, encapsulation DoE Study 1, (19 conditions/runs). Conditions presented in text two paragraphs before Figure 10; Supplement Table S3—Run conditions encapsulation DoE study 2 (16 conditions/runs). The factors studied and description of study runs in Figure 10; Supplement Table S4—Results fitting and analysis (encapsulation DoE study 2, model).
