# Supplementary material for: Commercialization of the Xalkori Pediatric Multiparticulate Product Using Quality-by-Design Principles
Source: Pharmaceutics. 2024 Aug 1;16(8):1027. doi: 10.3390/pharmaceutics16081027 (PMC11360164; doi:10.3390/pharmaceutics16081027)
Supplement: Supplementary file 1 [file pharmaceutics-16-01027-s001.zip › Supplemental tables for manuscript- 3105509.pdf]

Supplement Table S1 - Run conditions, MSC DoE (10 conditions/runs). The factors studied and description of study runs in figure 4.

Process Parameters Studied				
**Run**	**Disk Speed (rpm)**	**Extruder Temperature (°C)**	**Feed Rate (g/min)**	**Extruder Screw Speed (rpm)**
1	6500	75.0	200	175
2	6500	75.0	150	225
3	5500	75.0	150	175
4	5500	75.0	200	225
5	6000	82.5	175	200
6	5500	90.0	200	175
7	6500	90.0	150	175
8	5500	90.0	150	225
9	6500	90.0	200	225
10	6000	82.5	175	200

Supplement Table S2 - Run conditions, encapsulation DoE Study 1, (19 conditions/runs). Conditions presented in text two paragraphs before Figure 10.

Process Parameters Studied						
**Run**	**A: Air Knife Pressure (mbar)**	**B: Infeed Level (mm)**	**C: Fill Vacuum (mbar)**	**D: Fluidizing Air Flow Rate (L/min)**	**E: Fill Speed (CPH)**	**F: Air Puff Pressure (mbar)**
1	400	9.5	-250	20	14000	150
2	400	11.5	-150	20	26000	150
3	800	9.5	-250	20	14000	100
4	800	9.5	-250	26	14000	150
5	400	9.5	-150	26	14000	150
6	800	11.5	-150	26	14000	100
7	800	11.5	-150	26	14000	100
8	400	9.5	-150	26	14000	150
9	400	11.5	-250	20	14000	100
10	600	10.5	-200	23	20000	125
11	800	11.5	-150	20	14000	150
12	800	9.5	-150	20	26000	150
13	600	10.5	-200	23	20000	125
14	800	11.5	-250	20	26000	100
15	800	11.5	-250	26	26000	150
16	400	9.5	-250	26	26000	100
17	400	9.5	-250	26	26000	100
18	400	9.5	-150	20	26000	100
19	800	11.5	-250	20	26000	100

Supplement Table S3 - Run conditions encapsulation DoE study 2 (16 conditions/runs). The factors studied and description of study runs in Figure 10.

Process Parameters Studied			
**Run**	**A: Air Knife Pressure (mbar)**	**B: Infeed Level (mm)**	**C: Fill Vacuum (mbar)**
1	400	10.0	-170
2	300	9.0	-140
3	400	9.0	-170
4	600	9.0	-140
5	300	11.0	-200
6	600	11.0	-140
7	300	11.0	-140
8	300	9.0	-200
9	450	11.0	-190
10	350	11.0	-150
11	600	11.0	-200
12	400	11.0	-170
13	450	9.0	-150
14	400	10.0	-170
15	600	9.0	-200
16	350	9.0	-190

Supplement Table S4 - Results fitting and analysis (encapsulation DoE study 2, model)

Responses (unit)		Filled Capsule Weight RSD (%)
**Data Summary**	Mean	1.51
	Standard Deviation	0.1466
	Minimum	1.26
	Maximum	1.86
**Model Coefficients of Main Effects (*p-value*)**	Transformation	None
	Intercept	1.49
	A: Air Knife Pressure	0.0778 (*0.0077*)
	B: Infeed Level	0.0395 (*0.0529*)
	C: Fill Vacuum	0.0015 (*0.9433*)
**Model Coefficients of Interaction Effects (*p-value*)**	A*B	0.0680 (*0.0137*)
	A*C	0.1198 (*0.0006*)
**Model Coefficients of Quadratic Effects (*p-value*)**	A^2	0.3717 (*0.0053*)
	C^2	-0.3109 (*0.0161*)
**Fit Summary**	Adjusted R^2	0.8103
	Predicted R^2	0.5149
	RMSE	0.0639
